# Should the Approach to Pre-Procedural Cardiological Diagnostics in Patients with Peripheral Artery Disease Be Reconsidered? The Prevalence of Coronary Artery Disease in Asymptomatic Patients

**DOI:** 10.3390/jcm14248858

**Published:** 2025-12-15

**Authors:** Eugeniusz Hrycek, Gabriel Grzadziel, Magda Konkolewska, Edyta Halatek, Przemyslaw Nowakowski, Piotr Buszman, Krzysztof Milewski, Aleksander Zurakowski

**Affiliations:** 1 American Heart of Poland, Topolowa 16, 32-500 Chrzanów, Poland; 2 Department of Cardiology, Faculty of Medical Sciences, Andrzej Frycz Modrzewski Kraków University, 30-705 Kraków, Poland; 3 Faculty of Medical Sciences, University of Technology, Rolna 43, 40-555 Katowice, Poland; 4 American Heart of Poland, ul. Armii Krajowej 101, 43-316 Bielsko-Biała, Poland

**Keywords:** coronary angiography, coronary artery disease, Jeopardy Score scale, multilevel atherosclerosis, peripheral artery atherosclerosis

## Abstract

**Background/Objectives:** Patients with peripheral arterial disease (PAD) or carotid stenosis (CS) who do not exhibit symptoms suggesting the coexistence of CAD are often not evaluated for CAD. The aim of this study was to assess the prevalence of asymptomatic CAD identified by coronary angiography in patients with PAD or CS, undergoing peripheral arteriography, and without prior diagnosis of CAD. **Methods**: A total of 350 PAD patients undergoing peripheral angiography, without a history or symptoms of CAD were prospectively enrolled in this study. These patients underwent simultaneous coronary angiography during scheduled peripheral arteriography. The severity of CAD was assessed using the Jeopardy Score Scale (JSC) and by evaluating the number of major coronary vessels involved. **Results**: Significant coronary artery stenosis was detected in 52.86%, with 50.00% in the PAD group and 51.43% in the CS group. One-, two-, and three-vessel disease was present in 29.14%, 14.28%, and 10.01% of the study population, respectively. The JSC median for the entire cohort was 2 (0–4) and 4 (2–8) when CAD was diagnosed. The combination of CS and PAD was associated with the highest risk for CAD (73%), with a median JSC of 2 (0–8). **Conclusions**: The risk of co-occurrence of CAD in patients with PAD, regardless of the presence of CAD symptoms, is high, exceeding 50%. Consequently, (in accordance with the guidelines for the management of chronic coronary syndromes), in all patients with PAD, regardless of CAD symptoms, advanced cardiological diagnostics, including coronary CT angiography or functional imaging, should be considered.

## 1. Introduction

Patients with peripheral artery disease (PAD) represent a particularly vulnerable population prone to adverse cardiovascular events and are at a high risk of mortality due to coronary artery disease (CAD) [1,2,3]. Even asymptomatic PAD increases cardiovascular risk [3,4]. Risk factors for the development of both PAD and CAD are fundamentally the same and primarily include age, male gender, smoking, hypertension, diabetes, dyslipidemia, and renal insufficiency. Despite the high risk of CAD coexistence in this patient group, current clinical practice [2,4] does not recommend extensive cardiac diagnostic evaluation for patients without clear symptoms of coronary artery disease. Moreover, the latest ESC guidelines for the management of patients with PAD [3] do not provide clear recommendations regarding CAD diagnostics in this population. The ESC Peripheral Arterial & Aortic Diseases guidelines introduce the term polyvascular, which denotes the presence of atherosclerosis in different areas of vascularization. However, a unified, standardized diagnostic approach to polyvascular disease is currently lacking.

The aim of this study was to evaluate the prevalence of significant angiographic lesions in the coronary arteries of patients with peripheral arterial disease (PAD) who exhibited no clinical evidence of coronary artery disease (CAD). This study focuses on a cohort characterized by potentially low cardiac risk, who, under standard clinical conditions, would not typically be considered, even as candidates for non-invasive diagnostic assessment. This aspect constitutes a distinctive feature of the study’s design. The innovative aspect of the present study is the assessment of the prevalence of polyvascular disease in patients with PAD, based on systematic coronary angiography performed in individuals undergoing invasive evaluation of peripheral arteries.

## 2. Materials and Methods

The analysis prospectively included a group of PAD patients who did not exhibit typical angina symptoms according to the ESC definition [5], had unknown coronary artery anatomy, and no history of coronary revascularization in the 360 days preceding study inclusion. These patients were hospitalized between 2012 and 2016 at the American Heart of Poland Hospital in Chrzanow. During hospitalization, all patients provided written informed consent for both peripheral arteriography and coronary angiography. Each patient underwent a standard cardiological consultation, taking into account CAD/PAD risk factors (age, gender, smoking, diabetes, hypertension, hyperlipidemia, history of stroke or transient ischemic attack, renal insufficiency, family history, and any history suggestive of acute coronary syndrome without prior coronary angiography). The consultation also assessed the presence and severity of lower-limb and carotid artery atherosclerosis and evaluated angina symptoms in their medical history. Each patient underwent a resting 12-lead electrocardiogram (ECG) to identify changes suggestive of myocardial ischemia or prior myocardial infarction. Echocardiography was performed using standard views, primarily to evaluate left ventricular ejection fraction (LVEF) using the biplane Simpson method [4,5]. Based on these data, patients without angina symptoms or classical indications for advanced CAD diagnostics were considered eligible for further analysis. At the time the database was created, full access to patients’ medical records ensured a consistent dataset.

Systematic angiography was conducted in asymptomatic study group subjects because clinical practice indicates that this group is at high risk for coronary artery disease. Additionally, anginal symptoms are often difficult to detect due to coexisting intermittent claudication, which limits the capacity for physical exertion and may mask typical signs of ischemia. The coronary angiographies were performed according to the center’s standard diagnostic protocol in patients with PAD. It was performed during peripheral angiography, typically via radial access. Operators had access to the patients’ full medical histories (as required by the diagnostic process). As experienced interventional cardiologists, they posed a minimal risk of biased assessment. Hemodynamically significant stenosis was defined as a reduction in lumen diameter (Diameter Stenosis, DS) compared to the average of proximal and distal reference segments—≥50% for the left main coronary artery and ≥70% for all other epicardial arteries. Coronary angiography was additionally performed during peripheral arteriography solely for the purpose of pre-operative assessment of coronary anatomy, in accordance with the standard protocol of the documented department.

The severity of coronary artery disease was assessed using the Jeopardy Score (JSC) [6,7], which estimates the potential myocardial damage based on the location of coronary artery lesions and enables evaluation of the patient’s risk of cardiovascular mortality.

According to carotid stenosis evaluation, initial diagnosis was made using duplex Doppler ultrasound and CT angiography.

The aim of the study was to evaluate the prevalence of asymptomatic coronary artery disease (CAD) (as determined by coronary angiography) and its severity among patients with peripheral artery disease (affecting lower-limb and carotid arteries), undergoing peripheral arteriography, who had no previously known coronary artery anatomy or typical symptoms of angina pectoris. A prospective study employing systematic coronary angiography was conducted to evaluate the prevalence and extent of polyvascular disease in these patients.

Statistical analysis was performed using STATISTICA 12 PL (2623 Camino Ramon, Suite 200, San Ramon, CA 94583, USA), MedCalc 22.026 (MedCalc Software Ltd., Acacialaan 22, 8400 Ostend, Belgium), PQStat 1.8.4.142 (PQStat Software Grunwaldzka 591/B2, 62-064 Poznan–Plewiska, Poland), and Microsoft Excel. In all calculations, statistical significance was set at *p* < 0.05. For descriptive statistics, the mean and standard deviation (SD) were used as measures of central tendency and dispersion for parametric data. For nonparametric data, medians with upper and lower quartile in brackets were reported. As most of the dataset did not meet a normal distribution, the following nonparametric tests were utilized: Mann–Whitney test (to assess differences between two independent samples), Kruskal–Wallis test with post hoc analysis (to compare medians of three or more independent groups), and Spearman’s rank correlation coefficient (to assess correlations). Additionally, the Chi-square test (or Fisher’s exact test for 2 × 2 contingency tables) was utilized for the assessment of differences between proportions. The odds ratio (OR) was also employed as a relative measure of association, representing the ratio of the odds of an event occurring in one group to those in another group. Univariable and multivariable logistic regression analyses were performed to identify independent risk factors.

The study protocol conformed to the ethical guidelines of the 1975 Declaration of Helsinki. Approval from a bioethics committee or additional informed consent from the patient was not required, as all data were obtained through standard procedures routinely performed at the facility during hospitalization.

## 3. Results

**Figure 1:**
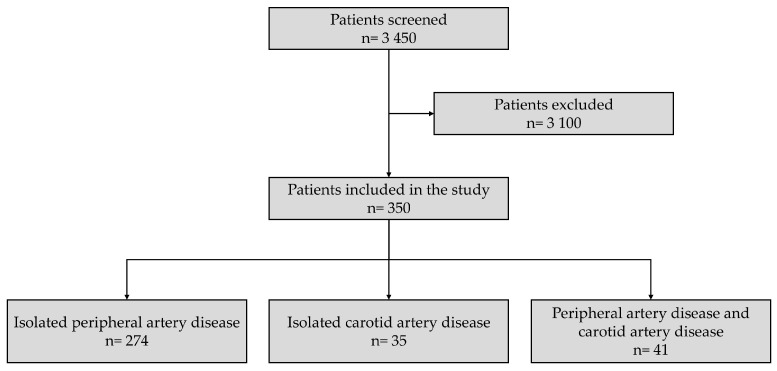
Patient selection flowchart.

From a total of 3450 patients hospitalized between 2012 and 2016 in The American Heart of Poland Vascular Surgery Department in Chrzanow, 350 met the inclusion criteria and were included in the study, with coronary angiography performed during planned peripheral arteriography (Figure 1). Demographic and clinical data are presented in Table 1.

None of the patients experienced typical anginal symptoms. Ischemic ST-T segment changes on ECG were noted in 51 patients (14.5%), and 31 patients (8.8%) had a history of myocardial infarction.

The largest group of patients included those with isolated PAD—274 (78.29%); those with isolated CS (carotid stenosis)—35 (10.00%); and those with coexisting PAD and CS—41 (11.71%). Significant coronary artery stenosis was diagnosed in 185 patients (52.86%), including 137 (50.00%) from the PAD population, 18 (51.43%) from the CS population, and 30 patients (73.17%) with both PAD and CS. Ultimately, a total of 138 patients (39.43%) were qualified for cardiac revascularization. Among these patients, 116 (33.14%) were qualified for percutaneous transluminal coronary angioplasty (PTCA), while 22 (6.28%) were qualified for coronary artery bypass grafting (CABG). Table 2 summarizes the angiographic data.

**Figure 2:**
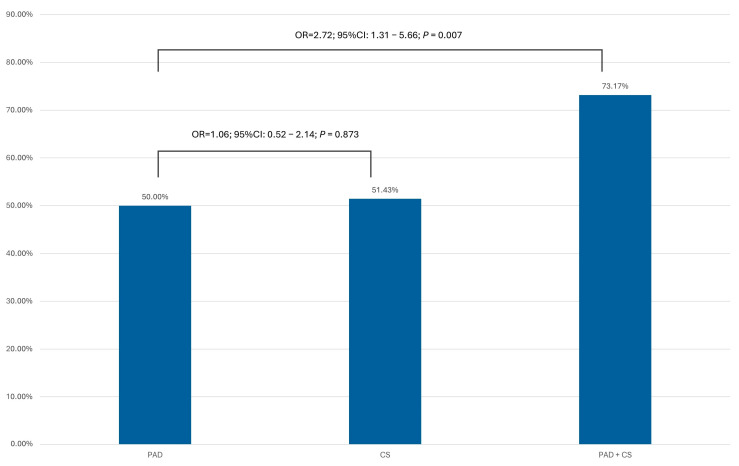
The comparison of the frequency of significant coronary artery disease in cardiac asymptomatic patients treated for PAD or CS. PAD—peripheral artery disease; CS—carotid artery disease.

A comparison of the frequency of significant coronary artery disease revealed a statistically significant difference between the PAD + CS and PAD subgroups but not in the PAD and CS groups (Figure 2).

**Figure 3:**
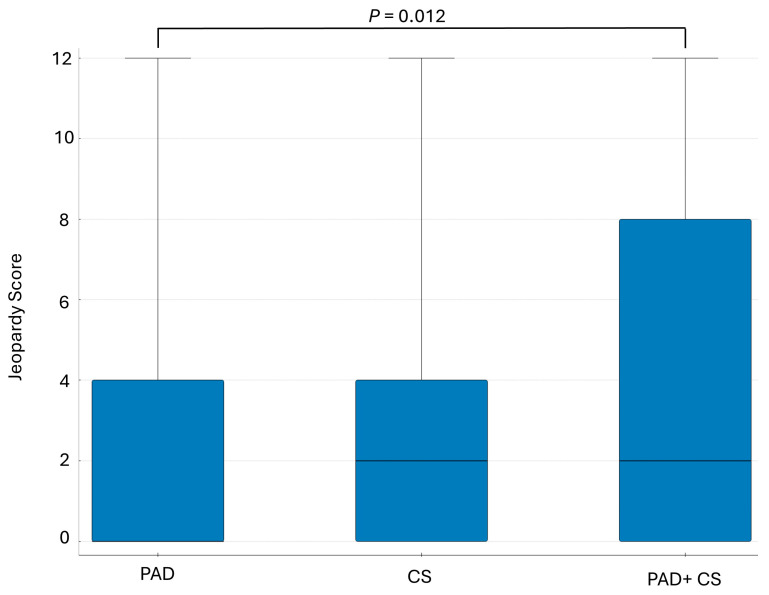
Comparison of the advancement of CAD (based on Jeopardy Score) in subgroups of patients with peripheral artery disease and carotid artery disease. PAD—peripheral arterial disease; CS—carotid artery disease.

The median JSC was 2 (0–4), with median scores of 0 (0–4) in the PAD group, 2 (0–4) in the CS group, and 2 (0–8) in the PAD + CS group. A Kruskal–Wallis test was conducted to compare the differences between the three patient groups. The results revealed a statistically significant difference between the groups (H = 8.30; *p* = 0.0156). Post-hoc analysis showed a statistically significant difference in JSC between the PAD and PAD + CS groups (Z = 2.88; *p* = 0.012) (Figure 3).

A comparison between the subgroup of individuals without stenocardial symptoms, with an LVEF of at least 50%, and no significant electrocardiographic changes (including ischemic or necrotic alterations, left bundle branch block, right bundle branch block, or left anterior fascicular block) (Group 1) and the remaining patients (Group 2) revealed that significant coronary artery stenosis was more frequent in Group 2 (61.29%) compared to Group 1 (43.29%) (OR: 0.48; 95% CI: 0.31–0.74; *p* = 0.001). Additionally, JSC was higher in Group 2, with a median of 2 (0–6), compared to Group 1, which had a median of 0 (0–2.5) (*p* = 0.0007). However, no significant difference was observed in the frequency of the maximum JSC level between Group 1 (11.26%) and Group 2 (7.02%) (*p* = 0.32).

Patients with a previous myocardial infarction (without prior invasive diagnostics) accounted for 8.86% of the study population (31 patients). Significant coronary artery stenosis was diagnosed in 27 of these patients (87.10%), with a median JSC of 6 (2–8).

Across all groups, patients with at least one significant coronary lesion had a similar CAD severity based on JSC—PAD group 4 (2–8); CS group 4 (2–10); and PAD + CS group 5 (2–9.5), with no statistically significant difference between the groups (*p* = 0.684). However, in the CS and PAD + CS populations, a higher proportion of patients had the maximum JSC score of 12 points (three patients—8.57% and four patients—9.76%, respectively), which was approximately three times higher than in the PAD group (nine patients—3.28%). These differences did not reach statistical significance.

In the studied population, a statistically significant, weak negative correlation was observed between LVEF and JSC (r = −0.207; *p* = 0.0001), and a weak positive correlation was observed between patient age and JSC (r = 0.148; *p* = 0.005). In univariable analysis, age, male sex, history of TIA and/or stroke, presence of arterial hypertension, presence of abnormalities in resting ECG, and LVEF < 50% were associated with the presence of significant coronary artery disease (Table 3, section A).

Multivariable logistic regression analysis revealed that male sex, the presence of arterial hypertension, ECG abnormalities, and reduced LVEF were independent risk factors for significant CAD. The strongest predictor of the presence of CAD was reduced LVEF (Table 3, section B).

## 4. Discussion

Patients with peripheral arterial disease (PAD) are individuals with a particularly unfavorable prognosis due to both the natural progression of the underlying condition and the high risk of coronary complications [8]. Considering the persistently high number of adverse cardiovascular events and the high prevalence of concomitant coronary artery disease (including asymptomatic cases), additional diagnostics appear justified [9,10]. Despite a higher mortality risk than in patients with coronary artery disease (CAD) after a myocardial infarction or stroke [11,12,13], there are currently no clear guidelines for diagnosing CAD in PAD populations.

Current guidelines for the treatment of patients with PAD suggest the utility of imaging studies for CAD but do not provide specific recommendations [3]. Similarly, the ESC guidelines for managing chronic coronary syndromes recognize PAD as a significant risk factor yet offer no clear diagnostic recommendations for CAD in this group of patients [4].

The population analyzed in the present research consists of patients who, due to the absence of angina symptoms, are often not considered for extensive cardiac diagnostics in typical clinical settings. This may be associated with increased risk of perioperative complications during vascular surgery and may contribute to the poor long-term prognosis observed in this patient group. The proportion of patients with significant coronary artery disease in the studied population was slightly lower compared with the results reported by Herz et al. [14] more than thirty years ago (52.9% vs. 59%). This difference is likely due to Herz including patients with a history of typical angina in his analysis; additionally, 30% of his patients had an abdominal aortic aneurysm. Similar results to those of the current study were reported by Sung Woo Cho et al. [9], who confirmed angiographically significant CAD in 62% of patients with PAD, of whom only 13% exhibited symptoms of CAD. The proportion of multivessel (at least two vessels affected) CAD in Sung Woo Cho’s study was higher (72% vs. 24.29%), likely due to the inclusion of patients with CAD symptoms [9]. On the other hand, within the present study, in the subgroup of patients without any coronary symptoms and without abnormalities in additional tests (ECG, echocardiography), the frequency of significant coronary artery stenosis exceeded 40%. Differences in the prevalence of CAD in the discussed studies may be caused by variations in patient symptomatology across the examined populations (symptomatic vs. asymptomatic individuals).

In the present study, patients with carotid artery disease (CS) exhibited an even higher risk of significant coronary artery stenosis (51.4% of patients), which was higher than the findings of Professor Illuminati’s team [15] (31% vs. 51.43%) but similar to the results of Hofmann R. [16] (61% vs. 51.43%) and David J. (64% vs. 51.43%) [17]. As a result, current guidelines for the treatment of this patient group recommend considering invasive coronary angiography, regardless of CAD symptoms.

An important and novel observation in the current study was the identification and analysis of a group of asymptomatic CAD patients burdened with both lower-extremity (PAD) and carotid artery atherosclerosis (CS). This group had not previously been studied regarding the frequency and severity of CAD based on non-invasive or angiographic examinations. In the present analysis, this group constituted 11.7% of the studied population, and the frequency of angiographically significant coronary artery disease exceeded 73%. The severity of CAD on the JSC scale was also the highest, reaching 5 (2–9.5) points. Furthermore, the highest 12-point score on the JSC scale was most commonly observed in this group. Consequently, the JSC results suggest that approximately 10% of patients in the PAD + CS subgroup may have an estimated five-year mortality risk of 45% [6]. Given this data, it may be reasonable to consider implementing comprehensive cardiac diagnostics, including routine coronary angiography, in this specific patient group. A novel approach in the present study, compared to previous works, was the use of the JSC scale to assess potential myocardial damage in case of a myocardial infarction due to vessel occlusion within the assessed lesions. This rapid and simple method allows for a more precise estimation of patient prognosis compared to the traditional classification into 1-, 2-, and 3-vessel CAD. The prognostic value of this indicator has also been confirmed in subsequent studies involving patients undergoing coronary revascularization [18]. The degree of coronary artery disease in the studied group was high, with a JSC median of 2 (0–4), and significantly increased in the presence of at least one coronary artery stenosis on coronary angiography. Based on this indicator, the five-year mortality risk for the whole population studied can be estimated at 3%, and for those with angiographically confirmed CAD, it can be estimated at 15% [6].

A comparison made between a subgroup of individuals without stenocardial symptoms, with an LVEF of at least 50%, and without significant electrocardiographic changes (such as ischemic or necrotic alterations, left bundle branch block, right bundle branch block, or left anterior fascicular block) (Group 1) and another subgroup comprising the remaining patients (Group 2) revealed that, as expected, both the prevalence and severity of coronary artery disease (CAD), assessed using the JSC scoring system, were higher in Group 2. Notably, even in Group 1, the prevalence of CAD exceeded 45%. Furthermore, no significant differences were observed between the groups in the frequency of the most severe JSC score of 12 points. These findings indicate that patients without clinical evidence of CAD but with some form of peripheral artery disease (PAD) may still be at an increased risk of significant CAD, including potentially advanced forms.

Importantly, when creating and assessing the utility of the JSC scale in estimating the prognosis of CAD patients, the presence of concomitant PAD was not considered. Taking into account the presence of PAD and its associated risk, the prognosis in the present study population may be significantly worse.

A key practical observation from this study is that approximately 40% of asymptomatic cardiac patients required revascularization. Importantly, the findings indicate that significant coronary artery disease requiring revascularization can occur even in patients with peripheral artery disease (PAD) and/or carotid stenosis (CS) who are considered to be at low cardiac risk. This underscores the need for enhanced efforts to identify such patients. Moreover, careful risk assessment is essential to avoid underestimating the likelihood of significant coronary disease in these patients. Future studies could consider exploring strategies to better identify these potentially at-risk individuals and evaluating the presence of polyvascular disease to assess its impact on outcomes in patients with PAD. It is also necessary to emphasize that clinical data about cardiac asymptomatic patients with PAD and/or CS remain limited.

In conclusion, taking these observations into account, and noting that patients with PAD may have an increased risk of CAD of around 40% according to ESC guidelines [4], this group could be considered as falling within the moderate or high (15–85%) pre-test likelihood of obstructive CAD. These findings may support considering additional diagnostic evaluation for CAD, including functional imaging or CCTA, even if resting ECG or echocardiography do not reveal abnormalities.

Based on the current data, it seems reasonable to consider performing coronary angiography in patients without prior comprehensive cardiological evaluation who are undergoing invasive peripheral angiography or percutaneous peripheral revascularization. In this scenario, the risk of complications is lower than in traditional coronary angiography (approximately 2% for classic coronary angiography [19]), due to the elimination of the most common local complications. In the presented study, one complication (0.28%), in the form of right coronary artery dissection requiring stent implantation, was noted.

An important limitation of the proposed approach is the lack of confirmation of myocardial ischemia based solely on coronary angiography. Detecting significant coronary lesions on angiography before planned vascular surgery raises the questions about the qualification for potential coronary revascularization. The assessment of the benefits of prophylactic coronary revascularization before planned surgeries in patients with PAD has been the subject of at least three randomized trials: (CARP) [20], DECREASE V [21], and the Systematic Strategy of Prophylactic Coronary Angiography Improves Long-Term Outcome After Major Vascular Surgery in Medium- to High-Risk Patients [22]. It should be emphasized that most of the studies conducted were performed before the widespread use of percutaneous coronary intervention procedures or were performed using first-generation metal or drug-eluting stents. Over the years, the criteria for qualifying patients for coronary revascularization have changed, and additional imaging and diagnostic methods for CAD, such as intravascular ultrasound (IVUS) or fractional flow reserve (FFR) assessment, have been introduced into clinical practice. These advances may improve both early and long-term outcomes of coronary angioplasty in this patient population.

A recently published study on the impact of coronary revascularization procedures on prognosis in patients with PAD demonstrated similar outcomes between PAD patients without CAD and those with CAD who underwent coronary revascularization [23]. The study indicated that a strategy of routine coronary angiography and subsequent PCI for significant CAD in symptomatic PAD patients undergoing PTA is safe and provides similar long-term survival compared to symptomatic PAD patients undergoing PTA without CAD. Additionally, the 5-year survival rate for the entire group was significantly higher (88.5%) than the survival rates reported in the literature for PAD patients (up to 50%) [24,25].

Performing routine coronary angiography in patients with peripheral arterial disease during arteriography remains a topic of debate. The question of how best to reduce early and late cardiovascular mortality in this patient population has not yet been definitively answered. One approach that could be considered is the routine non-invasive assessment of coronary arteries using angio-CT. While the detection of significant coronary artery stenosis in a patient with PAD does not automatically imply the need for coronary revascularization, it may help increase the awareness of the operating team during vascular procedures, as well as inform the patient and their outpatient physicians in long-term follow-up.

## 5. Conclusions

In conclusion, a strategy of routine coronary angiography, irrespective of CAD symptoms, with potential PCI in symptomatic PAD patients may be considered reasonable for those undergoing PTA or angiography. This approach helps detect significant coronary artery disease in a substantial percentage of patients, supporting the development of a suitable treatment plan, including intensive pharmacotherapy and, if needed, consideration for coronary revascularization. Such an approach may help improve the otherwise poor long-term prognosis in this patient group. Further randomized trials are needed to better understand the effectiveness and safety of this treatment approach for PAD patients [26]. The results obtained suggest that systematic coronary evaluation may be beneficial in the high-risk population studied.

## 6. Study Limitations

The most important limitation of this study is the single-center experience and the relatively small number of patients. The group of patients with both peripheral and carotid artery disease was limited to 41. The study protocol did not include the use of fractional flow reserve (FFR) or non-invasive ischemia testing in borderline cases. Angiographic assessment of coronary artery stenosis does not allow for evaluation of their functional significance, which may result in an overestimation of the number of clinically significant stenoses. It should be noted that results obtained may not be generalizable to populations with different risk profiles or screening strategies.

## Figures and Tables

**Table 1:** Demographic and clinical characteristics of the study group.

**Demographic Data (*n* = 350)**	
Men, *n* (%)	222 (63.43%)
Age, years	66 ± 8.5
Hypertension, *n* (%)	296 (84.57%)
Dyslipidemia, *n* (%)	146 (41.71%)
Type 2 diabetes on oral therapy, *n* (%)	77 (22.00%)
Type 2 diabetes on insulin therapy, *n* (%)	46 (13.14%)
Chronic kidney disease, *n* (%)	5 (1.43%)
Patients with a history of heart attack—at least one year before admission, *n* (%)	31 (8.86%)
Current smoking, *n* (%)	202 (57.71%)
Past smoking, *n* (%)	53 (15.14%)
Family history of cardiovascular disease, *n* (%)	79 (22.57%)
**Clinical Characteristics (*n* = 350)**	
Isolated peripheral artery disease (PAD), *n* (%)	274 (78.29%)
Isolated carotid artery disease, *n* (%)	35 (10.00%)
Peripheral artery disease and carotid artery disease, *n* (%)	41 (11.71%)
ST-T segment changes in resting ECG, *n* (%)	51 (14.57%)
Left ventricular ejection fraction (LVEF) (%)	60 (50–60)

**Table 2:** Analysis of angiographic data according to the location of coronary artery lesions, coexistence of peripheral artery disease, ECG changes, and echocardiographic findings.

**Number of Affected Vessels**	
Without significant coronary artery lesions, *n* (%)	163 (46.57%)
Single-vessel disease, *n* (%)	102 (29.14%)
Double-vessel disease, *n* (%)	50 (14.28%)
Multivessel disease, *n* (%)	35 (10.01%)
**Frequency of Significant Coronary Artery Lesions (>70% Diameter Stenosis) in the PAD, CS, and PAD + CS Subgroups**	
Location of atherosclerosis	Present stenosis (>70% DS, >50% LM)
Study group, *n* (%)	185 (52.86%)
PAD subgroup, *n* (%)	137 (50.00%)
CS subgroup, *n* (%)	18 (51.43%)
PAD + CS subgroup, *n* (%)	30 (73.17%)
**Presence of Significant Stenoses Depending on ECG Changes and Echocardiographic Examination**	
	Present stenosis (>70% DS, >50% LM)
Patients without symptoms, without changes in ECG and echocardiographic examination- Group 1 (*n* = 164), *n* (%)	71 (43.29%)
The rest of patients- Group 2 (*n* = 186), *n* (%)	114 (61.29%)

CS—carotid artery disease; DS—diameter Stenosis; ECG—electrocardiogram; PAD—peripheral artery disease; LM—left main coronary artery.

**Table 3:** Univariable (section A) and multivariable (section B) logistic regression models for the prediction of significant atherosclerotic lesions in coronary arteries in cardiologically asymptomatic patients with peripheral artery disease.

	(A) Univariable Logistic Regression Model				(B) Multivariable Logistic Regression Model			
	OR	−95% CI	+95% CI	*p*-Value	OR	−95% CI	+95% CI	*p*-Value
Age	1.350	0.880	2.061	0.165				
Sex	1.973	1.269	3.067	0.003	1.683	1.053	2.691	0.029
DM	1.435	0.911	2.26	0.119				
HA	2.141	1.177	3.894	0.013	1.990	1.048	3.781	0.035
Hyperlipidemia	1.04	0.679	1.592	0.857				
TIA or stroke	2.12	1.172	3.834	0.013	1.866	0.997	3.492	0.051
CKD	3.624	0.401	32.758	0.252				
Smoking	0.756	0.47	1.217	0.250				
Medical interview	1.324	0.798	2.197	0.278				
ECG changes	2.72	1.710	4.325	<0.0001	2.107	1.286	3.454	0.003
Reduced LVEF	3.875	1.86	8.073	0.0003	2.691	1.230	5.839	0.012

DM—type 2 diabetes; HA—arterial hypertension; TIA—transient ischemic attack; CKD—chronic kidney disease; LVEF—left ventricular ejection fraction.

## Data Availability

Derived data supporting the findings of this study are available from the corresponding author on request.

## Abbreviations

The following abbreviations are used in this manuscript:

CABG	Coronary Artery Bypass Grafting
CAD	Coronary Artery Disease
CCTA	Coronary Computed Tomography Angiography
CI	Confidence Interval
CKD	Chronic Kidney Disease
CS	Carotid Artery Disease/Carotid Stenosis
DM	Type 2 Diabetes Mellitus
ECG	Electrocardiogram
ESC	European Society of Cardiology
FFR	Fractional Flow Reserve
HA	Arterial Hypertension
IVUS	Intravascular Ultrasound
JSC	Jeopardy Score
LM	Left Main Coronary Artery
LVEF	Left Ventricular Ejection Fraction
OR	Odds Ratio
PAD	Peripheral Artery Disease
PCI	Percutaneous Coronary Intervention
PL	Polska/Poland
PTA	Percutaneous Transluminal Angioplasty
PTCA	Percutaneous Transluminal Coronary Angioplasty
SD	Standard Deviation
TIA	Transient Ischemic Attack
USA	United States of America
